# Effectiveness of Nutritional Advice for Community-Dwelling Obese Older Adults With Frailty: A Systematic Review and Meta-Analysis

**DOI:** 10.3389/fnut.2021.619903

**Published:** 2021-06-29

**Authors:** Yue-Heng Yin, Justina Yat Wa Liu, Tsz Man Fan, Kit Man Leung, Man Wai Ng, Tsun Yee Tsang, Ka Po Wong, Maritta Välimäki

**Affiliations:** ^1^School of Nursing, The Hong Kong Polytechnic University, Hong Kong, China; ^2^Xiangya Center for Evidence-Based Practice and Healthcare Innovation: A Joanna Briggs Institute Affiliated Group, Xiangya Nursing School, Central South University, Changsha, China; ^3^Department of Nursing Science, University of Turku, Turku, Finland

**Keywords:** nutritional advice, frailty, obesity, body weight, physical function

## Abstract

**Objectives:** This systematic review was aimed to examine the effectiveness of nutritional advise interventions compared with usual care, or exercise, or exercise combined with nutritional advice as a means of improving the body weight, body composition, physical function, and psychosocial well-being of frail, obese older adults.

**Methods:** CINAHL, Cochrane Library, Embase, MEDLINE, PsycINFO, and Scopus databases were searched to identify relevant studies. The quality of the included studies was assessed using Cochrane's risk of bias tool 2. Meta-analysis was performed with respect to body weight and fat mass. Other outcomes were synthesized narratively.

**Results:** Eight articles (from two studies) with a total of 137 participants were included in the review. The results revealed that nutritional advice was more effective than exercise in reducing body weight and fat mass. The nutritional advice was also beneficial in enhancing physical function and psychosocial well-being. However, it was less effective than exercise or combined interventions in increasing muscle strength and preventing lean mass loss.

**Conclusions:** Nutritional advice is an essential intervention for reducing body weight and fat mass, for enhancing physical function, and for improving the psychosocial well-being of obese older adults experiencing frailty. The limited number of studies included in this review suggests that there is a need for more well-designed interventional studies in order to confirm these findings.

## Introduction

Frailty, a well-known gerontological syndrome, is described as a decrease in resistance to stressors caused by multiple declines in physiological systems (1–4). Clinical manifestations of frailty include shrinking (unintentional weight loss or sarcopenia), weakness (low muscle strength or grip strength), poor endurance (self-reported exhaustion), slowness (slow walking speed), and low levels of physical activity (1, 2). Frailty can lead to multiple negative health outcomes, including unconsciousness, morbidity, institutionalization, falls, incontinence, and hospitalization (5). The prevalence of frailty among older people in 19 countries in Europe has been reported to be ~17% (6). A systematic review of 56 studies, with sample sizes ranging between 54 and 12,373, also observed that the pooled prevalence of frailty was around 17.4% among people of 60 years or older (7). The rapid increase in the number of frail older people worldwide has significantly increased the pressure on health care systems (8).

Although, obesity appears to be contradictory to frailty, it can also be found in frail older adults (9). The accumulation of fat mass and decreased muscle mass are an outcome of the physiological process of aging, which predisposes older adults to obesity (10, 11). Obesity in frail older adults can exaggerate physical inactivity and metabolic instability, which in turn contributes to frailty (12–14). Research in Spain reported that the prevalence of sarcopenic obesity and frail obesity among 1,765 non-institutionalized individuals was 17.2 and 4.0%, respectively, and that the numbers continue to grow (9). Given that obesity is becoming an epidemic, it is possible that obese older people will become the prevalent phenotype of frailty in future (15).

A limited number of studies have been conducted regarding interventions designed to address both frailty and obesity (3, 4, 13). These have mainly involved individual-level interventions, such as modifying lifestyle and social factors (4). Lifestyle interventions are common in community settings (4), wherein nutrition is deemed crucial to the postponement or reversal of frailty (16). The synergistic effects of various micro- and macronutrients within the whole diet are typically associated with the prevention and reversal of frailty (17). The modification of an individual's entire diet may represent an effective way of managing frailty. This can be achieved by providing nutritional advice, leading to a change in dietary behavior (18). In addition to nutritional supplements, nutritional advice has been used to help older adults establish healthy dietary habits. In the long term, sustainability of the effect of and compliance with such interventions could be improved (19).

The current search indicated that the effects of nutritional advice on the management of frail, obese older adults are yet to be adequately evaluated. Previous systematic reviews focused on either frail or obese older adults, without combining these two syndromes. The target interventions have been either exercise (20, 21) or nutritional supplements (22, 23), rather than the adoption of nutritional advice. However, nutritional advice remains an important method of implementing research findings into real practice. Therefore, the aim of this systematic review is to examine the effectiveness of nutritional advice compared with usual care, or exercise, or exercise combined with nutritional advice for managing body weight, body composition, physical function, and frailty-related physio-psychosocial parameters in obese, frail older adults. To this end, the following two research questions are addressed:

1. What are the components in nutritional advice interventions for obese, frail older adults?
2. How effective is nutritional advice compared with usual care, or exercise, or combined interventions in improving the body composition, muscle strength, physical function, and psychosocial well-being of obese, frail older adults living in the community?

## Methods

The Preferred Reporting Items for Systematic Reviews and Meta-Analysis (PRISMA) statements were employed in the reporting of this systematic review (24). The review protocol was registered in the PROSPERO database (CRD42019142403).

### Eligibility Criteria

The inclusion criteria were based on the population, intervention, comparator, outcome, and study (PICOS) approach (25):

1. Obese adults of 65 years or older in the community who meet at least three out of five criteria in the Fried's frailty phenotype (1) or other valid instruments, such as the Survey of Health, Aging, and Retirement in Europe (SHARE-FI) (26), pre-frail were not considered; obesity is defined as BMI or percentage of body fat with valid cutoff values.
2. All kinds of nutritional advice interventions aimed at combatting obesity and frailty; control groups which included participants who received a placebo or no treatment (passive control), exercise interventions (active control), or nutritional advice plus exercise interventions (combined control).
3. Primary outcomes included body weight, whereas, secondary outcomes included body composition indicators, such as bone mineral density (BMD), muscle mass, and fat mass, in addition to muscle strength, physical function (objective measures, including physical performance test, gait speed, balance), and psychosocial well-being.
4. Experimental studies, such as randomized controlled trials (RCTs), cluster RCTs, quasi non-equivalent control groups, and one group pre- and post-test studies.

Studies were excluded if the participants demonstrated any signs of chronic illness, had any comorbidities, such as diabetes, cancers, and cardiovascular diseases, or had been admitted to a hospital or nursing home. Furthermore, studies were excluded if the effects of the nutritional advice interventions were not separately reported. Observational studies, case reports, and commentaries were also excluded.

### Search Strategy

A systematic literature search was conducted on July 31, 2019 in the CINAHL, Cochrane Library, Embase, MEDLINE, PsycINFO, and Scopus databases without year limits. The search strategy was performed using the following keywords: (“obesity” OR “obese” OR “overweight”) AND (“frailty” OR “frail”) AND (“nutrition^*^ advice” OR “nutrition^*^ modification,” OR “nutrition^*^ intervention,” OR “nutrition^*^ counseling,” OR “nutrition^*^ guidance,” OR “nutrition^*^ education,” OR “diet^*^ therapy,” OR “diet^*^ advice,” OR “diet^*^ education,” OR “diet^*^ modification,” OR “diet^*^ counseling,” OR “diet^*^ guidance,” OR “lifestyle intervention,” OR “lifestyle modification”). The literature was limited to studies published in English. A hand search was also conducted of the reference lists of relevant papers retrieved from the database search.

### Study Selection

After duplicate articles were removed with the help of EndNote X8 software, the title and abstract of each paper were reviewed independently by two authors to exclude clearly irrelevant papers. Subsequently, the full-text versions of the articles were reviewed independently by the two authors. Any disagreements were resolved by discussing the relevant issues with another author.

### Data Extraction

Two authors extracted the data independently and then compared their findings. Any disagreements were solved through consultations with another author. The data extracted from each study included information on the title, author, year, country, demographic data (age, gender, settings, and diagnostic criteria of frailty); methodological data (sample size, group design, duration, and measurement timepoints); and outcome data (primary outcomes, including body weight, and secondary outcomes, including body composition, physical function, and psychosocial well-being). The outcome data were extracted in the form of mean values, standard deviations, and *P*-values. Furthermore, details of the components of the interventions were extracted in accordance with the template for intervention description and replication (TIDieR) checklist (27), which included the name of the intervention, the materials, procedures, provider, modes of delivery, location, dose, length, adherence, and the comparison group. In cases where parts of the data from the study was published in other articles, either the data from these other sources were extracted or contact was made with the authors if required.

### Risk of Bias in Individual Studies

The Cochrane tool for assessing the risk of bias in randomized trials (RoB 2 tool) was employed to assess the risk of bias in the studies (28). Five domains were assessed in each study, including the randomization process, intended interventions, missing outcome data, outcome measurements, and selection of the reported results. To determine whether the data were reported as planned, the study registration or protocol for each included study was obtained. Moreover, the quality of the included studies was evaluated independently by the two authors and disagreements were resolved through discussion with a third author. A funnel plot was not conducted due to the limited number of studies involved.

### Data Analysis and Synthesis

The descriptive characteristics of the studies were categorized manually. To compare the effects of nutritional advice interventions and combined controls, we pooled meta-analyses of the primary outcome (i.e., the body weight) and the secondary outcomes (i.e., the fat mass) using Review Manager 5.3 Software (29). In respect of the meta-analyses, it was found that the effect size using mean difference (MD) possessed 95% confidence intervals (95% CIs). Due to the varied nature of the study designs, a random-effects model was utilized. The heterogeneity of the studies was assessed using an I^2^ value of ≤50% as an indication of low heterogeneity (30). For outcomes not included in the meta-analyses (i.e., body composition, physical functioning, and psychosocial well-being), the information was synthesized narratively.

### Assessing the Quality of the Body of Evidence

The quality of the evidence was assessed using the Grading of Recommendations, Assessment, Development, and Evaluation (GRADE), which included five domains: risk of bias, imprecision, inconsistency, indirectness, and other factors, including publication bias (31).

## Results

### Study Selection

**Figure 1:**
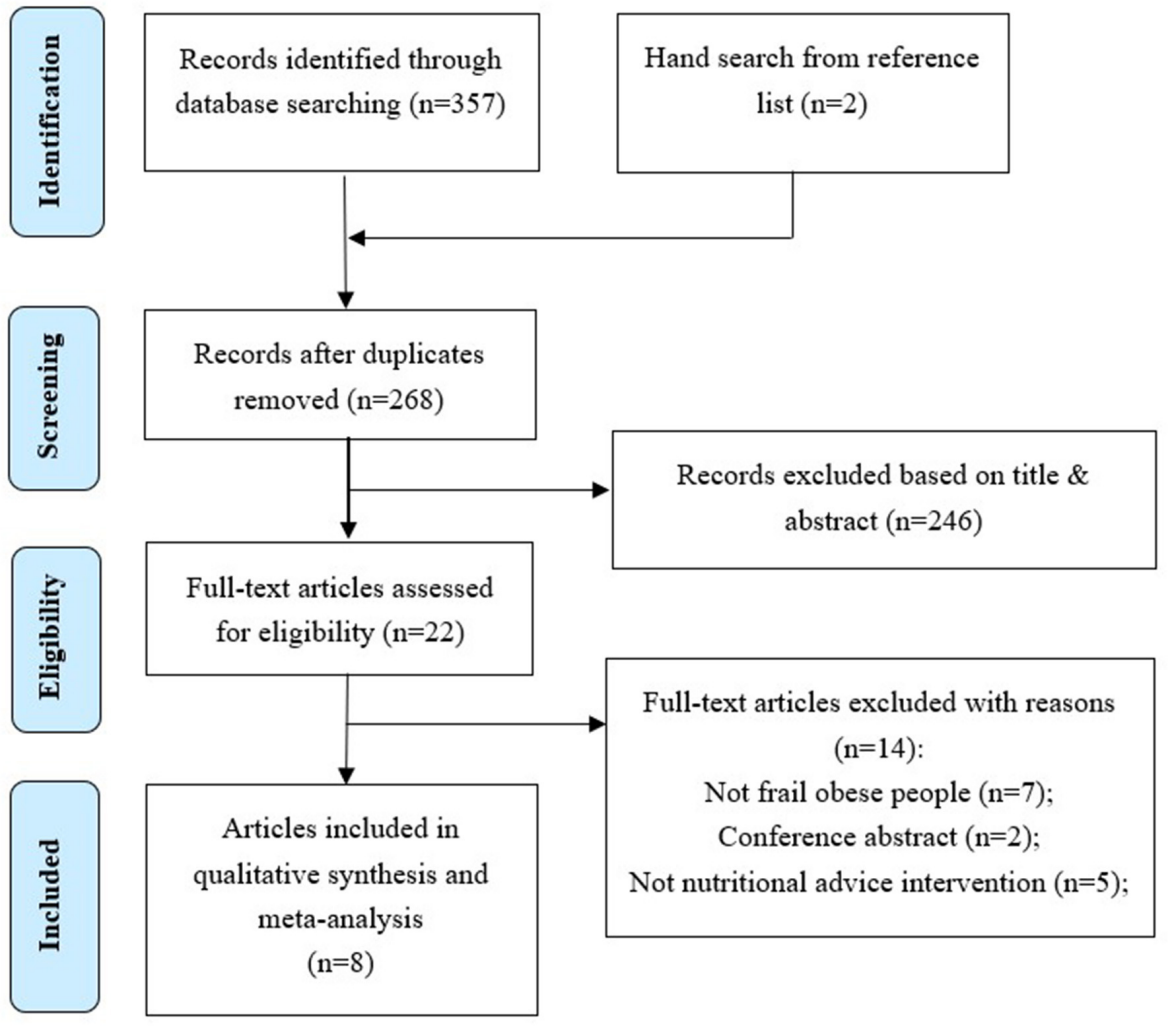
Flow chart of the study selection process.

The study selection process is indicated in Figure 1 and comprises a flow chart of the study selection process, which was implemented in accordance with the PRISMA flowchart (32). The literature search produced 357 hits. Two additional articles were identified through a hand search. After duplicates were removed, 268 hits remained. Once the titles and abstracts had been assessed, 245 hits were excluded. The full-text versions of the remaining 23 hits were reviewed. As per the review, another 15 articles were eliminated (see Appendix I), which left 8 articles (33–40). Since seven articles (33, 35–40) reported different findings from the same dataset, a total of two studies reported in eight articles have been included in this systematic review.

### Study Characteristics

The characteristics of the included studies are summarized in Table 1. The two included studies (33, 34) are both RCTs conducted in the United States. The sample size in the two studies was 137, comprising 30 and 107, respectively. Specifically, the combined studies consisted of 85 women and 52 men. The average age of the participants was 69.69 ± 4.08 years. The participants in both the studies presented with at least mild-to-moderate frailty and lived in the community.

**Table 1:** Characteristics of the included studies.

**Author, year**	**Country settings**	**Sample sizeGenderAge**	**Study type Duration**	**Group allocation (*n*)**	**Measurement time point**	**Outcomes**	**Findings**	**Frailty diagnostic criteria**	**Frailty level**
Villareal et al., (33)	USA Community	*n* = 107 37.4% male 70 ± 4.0	RCT 12 months	➀Diet advice (26) ➁Active control (exercise) (26) ➂Combined control (exercise + nutritional advice) (28) ➃Passive control (27)	Baseline; 6 months; 12 months	1) Body weight; 2) Body composition (fat mass, lean mass, BMD); 3) Physical function (total 1 RM, PPT, obstacle course, muscle strength, balance, mobility); 4) Quality of life; 5) Cognition	The combination of diet advice and exercise let to greater improvements in physical function, quality of life, and cognition than either intervention alone.	Two out of three operational criteria had to be met: 1) A score of 18–32 out of 36 on the modified physical performance test; 2) A level of 11–18 or 10–18 ml/kg of body weight per minute of peak oxygen consumption; 3) A self-report of difficulty in performing two instrumental activities of daily living or one basic activity of daily living.	Mild-to-moderate frailty
Frimel et al., (34)	USA Community	*n* = 30 40% male 70.3± 4.8	RCT 6 months	➀Diet advice (15) ➁Combined control (exercise + nutritional advice) (15)	Baseline; 6 months	1) Body weight; 2) Body composition (fat mass, lean mass); 3) Physical function (total 1RM, muscle strength)	Exercise combined with diet advice reduced the loss of muscle mass during voluntary weight loss and increased muscle strength in frail, obese older adults.		

*RCT, randomized controlled trial; BMD, bone mineral density; PPT, Physical Performance Test; 1 RM, one-repetition maximum*.

The two studies employed comparable diagnostic criteria for frailty, wherein participants were diagnosed with frailty if two out of three operational criteria were met. These criteria comprised: (1) a score of 18–32 out of 36 on Brown's modified physical performance test; (2) a level of 11–18 ml/kg of body weight/min of peak oxygen consumption; and (3) a self-report of difficulty in performing two instrumental activities of daily living or one basic activity of daily living (41).

### Risk of Bias Within the Studies

**Figure 2:**
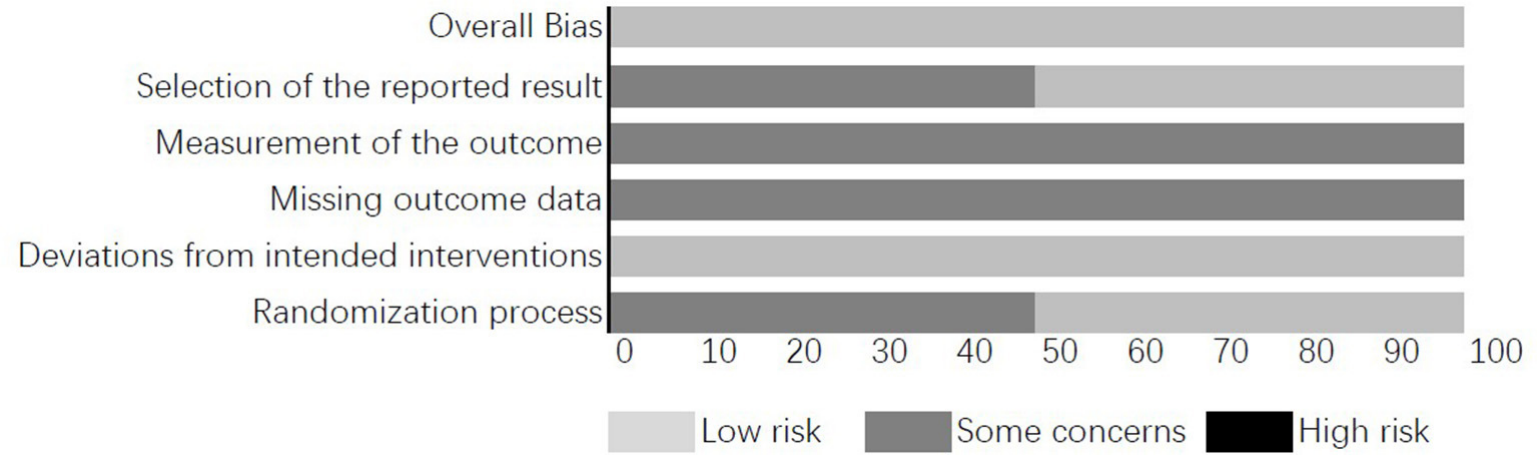
Assessment of the risk of bias in the included studies.

The quality of the included studies is shown in Figure 2, which outlines the assessment of the risk of bias in the included studies. The generation of random sequences and the concealment of the allocation were not described in detail in one study (34). The blinding of the participants was not clearly indicated in either study. However, it is not conceivable that the participants were unaware of the assigned groups. The blinding of the outcome assessments was performed well in both the studies by blinding the assessors to the group allocations. One study (33) reported the outcome data in alignment with the published protocol of the study. However, there was no discernable published protocol connected with the other study (34). Both the studies provided detailed information regarding the management of attrition data.

### Description of the Nutritional Advice Interventions

The interventions included in the two studies are summarized in Table 2. The duration of the interventions varied from 6 months (34) to 1 year (33). Villareal's (33) study was a four-armed design and included four groups, namely, nutritional advice, active control (exercise), combined control (nutritional advice + exercise), and passive control (no treatment). Frimel et al. 's (34) study consisted of two groups, to wit: nutritional advice and combined control.

**Table 2:** Description of the nutritional advice interventions of the included studies (modified based on the TIDieR checklist).

**Author**	**Brief name**	**Materials and procedures**	**BCTs**	**Providers and modes of delivery**	**Location, dose, and length**	**Fidelity and adherence**	**Comparison group**
Villareal et al., (33)	Diet advice	Weekly consultations. ➀A balanced diet of an energy deficit of 500–750 kcal/day + protein intake of 1 g/kg body weight/day. ➁Intake of supplements of 1,500 mg/day of calcium + 1,000 IU/day of vitamin D. ➂To achieve a weight loss of 10% of baseline body weight at 6 months and maintain it for another 6 months.	➀Set weekly behavioral goals; ➁Problem-solving; ➂Identification of high-risk situations; ➃Relapse prevention training.	Dietitian To review the food diaries and adjust the new caloric intake goals in weekly face-to-face meetings, and to help set weekly behavioral goals and conduct weigh-in sessions.	Location N/A Once per week, 12 months	Write a food diary 83% (interquartile range, 79–89)	Active Control: Three group training sessions per week of aerobic and resistance exercise. Each session was 90 min. Receive information on a healthy diet to maintain the current weight. Combined Control: Attended both diet and exercise group interventions. Passive Control: An intake of 1,500 mg/day of calcium + 1,000 IU/day of vitamin D, no advice related to diet or exercise, only received general information about a healthy diet during monthly visits.
Frimel et al. (34)	Diet advice	Weekly consultations. ➀A balanced diet of an energy deficit of 750 kcal/day (20% protein, 30% fat, 50% carbohydrates). ➁To achieve a weight loss of 10% body weight at 6 months with a weight loss of no more than 1.5% per week.		Dietitian To adjust caloric intake goals and hold weekly face-to-face meetings, and to provide behavioral change strategies.	Location N/A Once per week, 6 months	Write a food diary 100% (three people with poor compliance were excluded early on, the remaining 30 people completed the task)	Combined Control: Received the same diet intervention as diet group, but in a separate place. Attended exercise-training sessions as a group at an indoor exercise facility, which consisted of three 90-min sessions per week. Each session consisted of 15 min of flexibility exercises, 30 min of low-impact aerobic exercise, 30 min of high-intensity progressive resistance training, and 15 min of balance activities.

*BCTs, behavioral change techniques*.

In the nutritional advice interventions employed by the studies, the participants in Villareal et al. 's (33) research were asked to achieve an energy deficit of 500–750 kcal/day from their daily energy requirement and to take supplements of 1,500 mg of calcium/day and 1,000 IU of vitamin D/day. The participants in another study (34) were asked only to maintain an energy deficit of 750 kcal/day and to follow the recommendation that their diet consist of 20% protein, 30% fat, and 50% carbohydrates. The methods used to deliver the nutritional advice interventions in both the studies involved weekly group meetings with dietitians in order to obtain individual dietary prescriptions, weigh-in sessions, and consultations. The consultation sessions included problem-solving, the identification of high-risk situations, and relapse prevention training. The participants in both studies were required to record their food consumption in food diaries, which were reviewed weekly. In addition, the two studies had the same weight loss goal, which was to lose 10% of baseline body weight at 6 months. New goals were set weekly based on individual diary reports.

### Effects of the Nutritional Advice Interventions on Primary Outcomes

**Figure 3:**
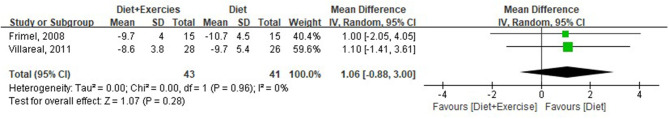
Forest plot of the effects of dietary advice with or without exercise on body weight.

Body weight fell significantly (a 10% decrease from baseline at 12 months) in the intervention groups after the receipt of nutritional advice, compared with the results of the passive control groups. Nutritional advice, either solely or combined with exercise, was proved to be more effective in reducing body weight than exercise alone. There were no significant differences between the effects of nutritional advice with or without exercise on reducing body weight revealed in the meta-analysis (*P* = 0.28, Figure 3: Forest plot of the effects of dietary advice with or without exercise on body weight). We have moderate confidence in the estimate of the effect (see Table 3: GRADE summary).

**Table 3:** Summary of GRADE for nutrition advice compared with the combined control among trials.

	**Certainty assessment**							**No. of patients**		**Effect**		**Certainty**
	**No. of studies**	**Study design**	**Risk of bias**	**Inconsistency**	**Indirectness**	**Imprecision**	**Other considerations**	**Nutrition advice**	**Combined control**	**Relative (95% CI)**	**Absolute (95% CI)**	
Body weight	2	Randomized trials	Serious^a^	Not serious	Not serious	Not serious	None	43	41	—	MD 1.06 higher (0.88 lower to 3 higher)	⊕⊕⊕◯ Moderate
Fat mass	2	Randomized trials	Serious^a^	Not serious	Not serious	Not serious	None	43	41	—	MD 0.13 higher (1.5 lower to 1.76 higher)	⊕⊕⊕◯ Moderate

*CI, confidence interval; MD, mean difference*.

a *There were some concerns about the randomization process, the report of the results, and deviations from the intended interventions*.

### Effects of the Nutritional Advice Interventions on Secondary Outcomes

**Figure 4:**
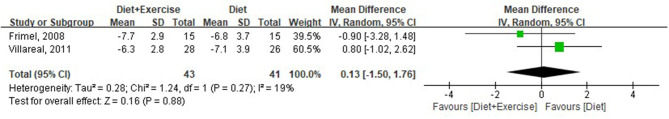
Forest plot of the effects of dietary advice with or without exercise on fat mass.

Secondary outcomes include body composition, muscle strength, physical function, and psychosocial well-being. One study (33) reported that nutritional advice significantly reduced fat mass (a 17% decrease in 12 months), including thigh, visceral, trunk, and subcutaneous fat, in the intervention group when compared with the passive control group. In the 12 months following the nutritional advice intervention, there was also a slight reduction in lean mass and BMD, comprising a 5% decrease and 3% decrease, respectively (33). Compared with nutritional advice, exercise alone or combined with nutritional advice was capable of lessening the reduction in lean mass (nutritional advice: exercise: combined = −5%: 2%: −3%) and BMD (nutritional advice: exercise: combined = −3%: 1%: −1%), while reducing fat mass (33). This result was supported by the other study (34), in which the combined control group lost less lean mass (upper extremity: 0.1 ± 0.2 vs. 0.2 ± 0.2 kg, *P* = 0.03; lower extremity: 0.9 ± 0.8 vs. 2.0 ± 0.9 kg, *P* =0.001) and fat-free mass (1.8 ± 1.5 vs. 3.5 ± 2.1 kg; *P* = 0.02) compared with the group that received dietary advice alone. There was no significant difference between the effects of dietary advice and combined control in terms of reducing fat mass, as demonstrated by the meta-analysis (*P* = 0.88, Figure 4: Forest plot of the effects of dietary advice with or without exercise on fat mass). We have moderate confidence in the effect estimate (Table 3: GRADE summary).

It can be inferred that exercise is more effective than nutritional advice in improving muscle strength (strength of knee extension and flexion: 25 vs. 0%) and gait speed (11 vs. 5%) (33). Physical function, as determined by the physical performance test (12 vs. 1%, *P* < 0.001) and the balance test (the one-leg stance test: 40 vs. −20%, *P* = 0.001), had significantly improved in the group that received nutritional advice, compared with the passive control group at 12 months (33). There were also greater improvements in physical function and muscle strength among those offered with a combination of nutritional advice and exercise than those offered with only nutritional advice, as seen in the results from the following tests: the physical performance test [19 vs. 12%, *P* < 0.001 (33)] the total one-repetition maximum test [30 vs. 0%, *P* < 0.001 (33); 43 vs. 0%, *P* < 0.05 (34)], the strength of knee extension test [20 vs. 0% (33); 33 vs. 10%, *P* = 0.04 (34)], the strength of flexion test [21 vs. 0% (33); 19 vs. 10%, *P* = 0.02 (34)], the one-leg stance test [75 vs. 40%, *P* = 0.18 (33)], and the gait speed test [23 vs. 5%, *P* = 0.04 (33)].

Psychosocial well-being was examined in only one study (33). This was achieved by measuring the quality of life (the Impact of Weight on Quality of life-Lite test), mood (the Geriatric Depression Scale score), and cognition change (the Modified Mini-Mental State test). The study in question stated that over a 12-month period, nutritional advice led to significant improvements in the quality of life and cognition, consisting of increases of 9 and 2%, respectively. However, there was no significant change in mood (*P* = 0.78). In addition, over a 12-month period, the impact of the exercise intervention on quality of life and cognition was greater than the impact of nutritional advice intervention, resulting in an increase of 13% (*P* = 0.001) and 3% (*P* = 0.0001), respectively (33). Meanwhile, the group that received nutritional advice combined with exercise showed the greatest improvements in quality of life, mood, and cognition among the four groups. The other three were the nutritional advice group, the exercise group, and the passive control group (33).

## Discussion

The aim of this systematic review was to examine the effectiveness of nutritional advice compared with usual care, or exercise, or exercise combined with nutritional advice intervention. A total of two studies published in eight articles and involving 137 participants were included in this review. The components of the nutritional advice intervention in both the studies emphasized the importance of delivering nutritional knowledge tailored to individual circumstances, the provision of continuous consultations, and the gradual attainment of nutritional objectives, typically within a period of between 6 and 12 months. The results of the narrative synthesis and meta-analyses indicated that nutritional advice interventions were beneficial in reducing body weight and fat mass, improving physical function and muscle strength, and enhancing psychosocial well-being. The results also indicated that the combined effects of exercise and nutritional advice were more prominent than either nutritional advice or exercise alone. However, the findings need to be treated with caution due to the limited number of studies and participants involved.

### Components of Nutritional Advice Interventions

The dietary recommendations in the two studies are worthy of consideration, not least with regard to the goal of weight reduction (a 10% decrease from the baseline weight in 6 months), which occurred at a moderate and steady pace. Thus, the two studies revealed beneficial long-term effects in this population. Furthermore, the energy deficit range is also thought-provoking. Currently, there are no standardized requirements regarding caloric restrictions for this population since this group needs to increase their intake of nutrients to fend off the possibility of frailty, while limiting their intake of calories in order to reduce obesity. Any form of “simple” weight loss intervention, whether intentional or not, may generate negative outcomes by reducing muscle mass and bone density (42–45). Since these two studies were conducted by the same research team, the goal of caloric restriction was adjusted to be smaller in Villareal's study (33) in comparison to the other study (34), that is, to an energy deficit of 500–750 kcal/day.

The delivery of the nutritional advice interventions in both studies also deserved some attention. The weekly group meetings, which comprised individual consultations, demonstrated a positive influence on weight control efforts. The “food record” and “weigh-in” sessions also helped the participants to persist in pursuing their goals. One published systematic review found that the effectiveness of intervention increased when a specific cluster of self-regulatory techniques was also employed, including goal-setting and self-monitoring (46). Nevertheless, this self-reported method has also generated considerable controversy regarding its veracity, principally because participants tend to report their food consumption as being close to the desirable value (47).

It is possible that the above common components of nutritional advice were critical to generating positive health outcomes. However, because the number of studies included was limited, the optimal components, and duration of intervention for obtaining beneficial effects on weight control or psychosocial well-being remain unclear.

### Effects of Nutritional Advice Interventions

The results of this review indicated that nutritional advice followed by a calorie-restricted diet (for an energy deficit of 500–750 kcal/day) is more effective than an exercise intervention alone in reducing body weight and fat mass. Conversely, a loss in lean mass and a decrease in BMD could not be avoided, even when a diet involving the intake of high-quality protein (1 g/body weight kg/day), calcium (1,500 mg/day), and vitamin D (1,000 IU) was prescribed. The combined effects of nutritional advice and exercise were not superior to nutritional advice alone in reducing body fat. However, nutritional advice combined with exercise was able to prevent the loss of lean mass. This finding was supported by another review, which highlighted the impact of dietary interventions combined with exercise on maintaining muscle mass in obese people (48).

In addition to reducing body weight and fat mass, nutritional advice interventions also played an important role in improving physical function. It is believed that there is a strong correlation between changes in physical function and the loss of fat mass (49, 50). Physical function, in terms of walking speed, balance ability, and mobility, is susceptible to the damage created by the age-related increase in fat mass, which can then lead to physical frailty (51, 52). Considering the prominent effects of nutritional advice interventions on reducing fat mass, it was not surprising to see that the nutritional advice interventions led to significant improvements in balance ability. Nevertheless, with regard to muscle strength, the effects of exercise were more prominent. Nutritional advice alone appears to have only a limited positive impact on muscle strength. The results of nutritional studies remain equivocal (53), and the number of studies in this area is still limited. Specific nutritional recommendations, such as those pertaining to the extent of caloric restrictions and nutritional intake, require further exploration. In addition, these findings may shed some light on the clinical health care among older people facing potential medical complications associated with obesity and consequent frailty, and provide evidence for nutrition guidance in community health care settings.

However, the effects of nutritional advice on changes in mood and cognition were only evident when combined with exercise. The positive effects of exercise on mood have also been reported in previous articles (54, 55). This may be because exercise can induce the release of endorphins, which regulate mood and cognition by activating opioid receptors (56).

### Limitations

There were some limitations in this systematic review. First, only studies published in English were included. The language limitation may mean that some relevant studies were omitted. Second, because of the limited scope of what could be considered, it is possible that a more wide-ranging and less-precise search strategy could have turned up more trials of relevance to this area of study. However, we believe that it is unlikely that we have overlooked any large relevant trials. We have searched PROSPERO but found no similar ongoing reviews. In addition, we searched in ClinicalTrials.gov and found that there were no ongoing interventional studies specifically related to nutrition advice in managing frail obesity. The landscape of knowledge may not change in the coming years, and therefore more studies in this area are urgently needed.

## Conclusion

This systematic review indicates that nutritional advice is an essential intervention in any attempt to reduce body weight and fat mass and to enhance the physical function of obese older adults experiencing frailty. However, nutritional advice in combination with exercise can lead to enhanced preservation of lean mass and BMD in addition to further improving muscle strength and psychosocial well-being. It is essential that nutritional advice aimed at obese older individuals experiencing frailty should be tailored to the specific physiological characteristics of this population, because caloric restriction needs to be moderate and other nutrients, such as protein or calcium, also need to be supplemented.

More well-designed, in-depth studies focusing on nutritional advice are needed to verify these findings and to identify the optimal caloric restriction range and duration of the dietary intervention required to achieve long-term effects on body composition and psychosocial well-being. The mode of combining nutritional advice with different exercise types in this population also requires further exploration.

## Data Availability Statement

The original contributions presented in the study are included in the article/Supplementary Material, further inquiries can be directed to the corresponding author/s.

## Author Contributions

The original idea for the study came from JL who guided the whole process. The articles were initially screened by TMF and KML. MWN and TYT participated in the data extraction process. TYT and KPW participated in the risk assessment process. YY-H and MV drafted the manuscript. All of the authors gave their final comments and approved the submission of this article to the journal.

## Conflict of Interest

The authors declare that the research was conducted in the absence of any commercial or financial relationships that could be construed as a potential conflict of interest.
