# Supplementary material for: Effectiveness of Nutritional Advice for Community-Dwelling Obese Older Adults With Frailty: A Systematic Review and Meta-Analysis
Source: Front Nutr. 2021 Jun 29;8:619903. doi: 10.3389/fnut.2021.619903 (PMC8275925; doi:10.3389/fnut.2021.619903)
Supplement: Supplementary file 1 [file Table_1.docx]

**Appendix Ⅰ: Reasons for excluding full-text articles**

| No. | Reference | Reasons for exclusion |
| --- | --- | --- |
| 1 | Shah K, Armamento‐Villareal R, Parimi N, Chode S, Sinacore DR, Hilton TN, et al. Exercise training in obese older adults prevents increase in bone turnover and attenuates decrease in hip bone mineral density induced by weight loss despite decline in bone‐active hormones. Journal of Bone and Mineral Research. 2011;26(12):2851-9. | Participants were not frail, only obese. |
| 2 | Serra-Prat M, Terradellas M, Arus M, Salietti A, Sanchez L, Ramirez S, et al. Effect of a nutritional and physical exercise intervention on changes in body composition and risk of frailty in elderly obese subjects. European geriatric medicine. 2019;10:S21‐. | Conference abstract |
| 3 | Porter Starr KN, Pieper CF, Orenduff MC, McDonald SR, McClure LB, Zhou R, et al. Improved Function With Enhanced Protein Intake per Meal: a Pilot Study of Weight Reduction in Frail, Obese Older Adults. Journals of gerontology Series A, Biological sciences and medical sciences. 2016;71(10):1369‐75. | Participants have renal problems. |
| 4 | Nct. Diet and Exercise Plus Metformin to Treat Frailty in Obese Seniors. <https://clinicaltrialsgov/show/NCT04221750>. 2020. | Not complete trial, and the participants were with diabetes, not only frail obesity. |
| 5 | Nct. Efficacy of an Intervention to Prevent Frailty in Obese Elderly People (PRE-FROB). <https://clinicaltrialsgov/show/NCT03000907>. 2016. | The target participants were not frail. |
| 6 | Nct. Lifestyle Intervention and Testosterone Replacement in Obese Seniors. <https://clinicaltrialsgov/show/NCT02367105>. 2015. | Drug was used, no sole nutrition advice interventions. |
| 7 | Mimi Tse MY, Peony Lai WY, Rose Heung SM, Iris Benzie FF. Overweight and obesity among community-dwelling older adults: Health-related issues and treatment. Overweightness and Walking2010. p. 211-22. | Participants were with diabetes, not only frail obesity. |
| 8 | Shah K, Parimi N, Chode S, Sinacore D, Napoli N, Armamento-Villarea R, et al. Effects of weight loss, exercise, or combined on bone mineral density and markers of bone turnover in frail obese older adults. Journal of bone and mineral research. 2010;25:S76‐. | Conference abstract |
| 9 | Xu F, Delmonico MJ, Lofgren IE, Uy KM, Maris SA, Quintanilla D, et al. Effect of a Combined Tai Chi, Resistance Training and Dietary Intervention on Cognitive Function in Obese Older Women. The Journal of frailty & aging. 2017;6(3):167-71. | Combined intervention, no sole nutrition advice interventions. |
| 10 | Villareal DT, Aguirre L, Gurney AB, Waters DL, Sinacore DR, Colombo E, et al. Aerobic or Resistance Exercise, or Both, in Dieting Obese Older Adults. New England journal of medicine. 2017;376(20):1943‐55. | No sole nutrition advice aimed at combatting obesity and frailty was used. |
| 11 | Villareal DT, Banks M, Sinacore DR, Siener C, Klein S. Effect of weight loss and exercise on frailty in obese older adults. Archives of Internal Medicine. 2006;166(8):860-6. | Only combined intervention, no sole nutrition advice was used. |
| 12 | Villareal DT, Shah K, Banks MR, Sinacore DR, Klein S. Effect of weight loss and exercise therapy on bone metabolism and mass in obese older adults: a one-year randomized controlled trial. Journal of clinical endocrinology and metabolism. 2008;93(6):2181‐7. | Only combined intervention, no sole nutrition advice was used. |
| 13 | Porter Starr KN, McDonald SR, Jarman A, Orenduff M, Sloane R, Pieper CF, et al. Markers of Renal Function in Older Adults Completing a Higher Protein Obesity Intervention and One Year Later: findings from the MEASUR-UP Trial. Journal of nutrition in gerontology and geriatrics. 2018;37(2):117‐29. | Participants have renal problems. |
| 14 | Porter Starr KN, Orenduff M, McDonald SR, Mulder H, Sloane R, Pieper CF, et al. Influence of Weight Reduction and Enhanced Protein Intake on Biomarkers of Inflammation in Older Adults with Obesity. Journal of nutrition in gerontology and geriatrics. 2019;38(1):33‐49. | Participants have renal problems. |
